# Telomere Length in Chromosomally Normal and Abnormal Miscarriages and Ongoing Pregnancies and Its Association with 5-hydroxymethylcytosine Patterns

**DOI:** 10.3390/ijms22126622

**Published:** 2021-06-21

**Authors:** Mikhail I. Krapivin, Andrei V. Tikhonov, Olga A. Efimova, Anna A. Pendina, Anna A. Smirnova, Olga G. Chiryaeva, Olga E. Talantova, Lubov’ I. Petrova, Vera S. Dudkina, Vladislav S. Baranov

**Affiliations:** 1 D.O. Ott Research Institute of Obstetrics, Gynecology and Reproductology, Mendeleevskaya Line 3, 199034 Saint Petersburg, Russia; krapivin-mihail@mail.ru (M.I.K.); tixonov5790@gmail.com (A.V.T.); pendina@mail.ru (A.A.P.); chiryaeva@mail.ru (O.G.C.); olga_talantova@mail.ru (O.E.T.); petrovaluba@mail.ru (L.I.P.); dudkinavs@mail.ru (V.S.D.); baranov@vb2475.spb.edu (V.S.B.); 2 Department of Medical Biophysics, Saint Petersburg State Pediatric Medical University, Litovskaya Street 2, 194100 Saint Petersburg, Russia; smirnova.ann551@yandex.ru

**Keywords:** telomeres, chorionic cytotrophoblast, pregnancy, miscarriage, telomere length, 5-hydroxymethylcytosine, epigenetics, heredity

## Abstract

The present study investigates telomere length (TL) in dividing chorionic cytotrophoblast cells from karyotypically normal and abnormal first trimester miscarriages and ongoing pregnancies. Using Q-FISH, we measured relative TLs in the metaphase chromosomes of 61 chorionic villous samples. Relative TLs did not differ between karyotypically normal samples from miscarriages and those from ongoing pregnancies (*p* = 0.3739). However, among the karyotypically abnormal samples, relative TLs were significantly higher in ongoing pregnancies than in miscarriages (*p* < 0.0001). Relative TLs were also significantly higher in chorion samples from karyotypically abnormal ongoing pregnancies than in those from karyotypically normal ones (*p* = 0.0018) in contrast to miscarriages, where relative TL values were higher in the karyotypically normal samples (*p* = 0.002). In the karyotypically abnormal chorionic cytotrophoblast, the TL variance was significantly lower than in any other group (*p* < 0.05). Assessed by TL ratios between sister chromatids, interchromatid TL asymmetry demonstrated similar patterns across all of the chorion samples (*p* = 0.22) but significantly exceeded that in PHA-stimulated lymphocytes (*p* < 0.0001, *p* = 0.0003). The longer telomere was predominantly present in the hydroxymethylated sister chromatid in chromosomes featuring hemihydroxymethylation (containing 5-hydroxymethylcytosine in only one sister chromatid)—a typical sign of chorionic cytotrophoblast cells. Our results suggest that the phenomena of interchromatid TL asymmetry and its association to 5hmC patterns in chorionic cytotrophoblast, which are potentially linked to telomere lengthening through recombination, are inherent to the development programme. The TL differences in chorionic cytotrophoblast that are associated with karyotype and embryo viability seem to be determined by heredity rather than telomere elongation mechanisms. The inheritance of long telomeres by a karyotypically abnormal embryo promotes his development, whereas TL in karyotypically normal first-trimester embryos does not seem to have a considerable impact on developmental capacity.

## 1. Introduction

Telomeres are dynamic nucleoprotein structures consisting of DNA tandem repeats 5′-TTAGGG-3′, shelterin proteins and telomere repeat-containing RNA (TERRA) [1,2,3,4,5]. Telomeres cap the ends of linear chromosomes, protecting them from degradation by endonucleases and end-to-end fusions [6,7,8]. Telomere length (TL) in a chromosome is determined by the number of tandemly repeated hexanucleotides and depends on the balance between the mechanisms of telomere attrition and lengthening. Telomeres shorten with each cell division cycle because of the incomplete DNA replication of linear chromosomes by DNA polymerase [9]. Apart from mitotic activity, telomere shortening may be caused by several other factors, including DNA damage, inflammation and oxidative stress [10,11,12]. Telomere lengthening mechanisms include telomerase enzymatic activity [13] and the recombination-based alternative lengthening of telomeres (ALT) [14,15,16]. In the absence of telomere-lengthening mechanisms, a cell that has undergone a certain number of replication rounds or adverse exposures enters senescence frequently accompanying by chromosomal instability and chromothripsis [17,18] and, eventually, apoptosis. Therefore, adequate telomere length regulation is the sine qua non of a cell’s normal functioning.

Substantial evidence has been found to support the association of anomalous telomere length changes in humans with the development of various pathological conditions, including cancer [19,20,21,22], cardiovascular diseases [23,24], diabetes mellitus [25] and neurodegenerative disorders [26,27]. Studies also confirm the role of telomeres in the realisation of the reproductive function. Both maternal and paternal telomere shortening may contribute to idiopathic recurrent pregnancy loss [28]. Shortened telomeres in sperm are sometimes observed in cases of idiopathic male infertility [29]. Shorter telomeres in oocytes and blastomeres are associated with the development of oocyte aneuploidy [30]. Women of advanced maternal age with shorter telomeres run a higher risk of conceiving a child trisomic for chromosome 21 than their counterparts with longer telomeres [31].

During embryogenesis, cells of the embryo and extraembryonic membranes undergo intensive divisions to gradually form and supply all types of body cells and tissues. Therefore, maintaining a certain telomere length at this stage is a necessary prerequisite to the embryo’s normal development. Few studies investigate cause-and-effect relations between telomere length alterations and post-implantation human embryonic development disorders. Most of them deal with placental telomere shortening in stillbirths, placental dysfunction in preeclampsia and intrauterine growth restriction—in other words, disorders of advanced gestational age [32,33,34]. By contrast, the present study focuses on investigating TL in the first trimester of gestation—a period marked by a robust natural negative selection of embryos with both anomalous and normal karyotypes, which results in 15% of pregnancies ending in miscarriage. We assessed TLs in chorion from first-trimester miscarriages and ongoing pregnancies, factoring in the presence of chromosome abnormalities. Furthermore, considering the role of epigenetic mechanisms in telomere regulation, we investigated whether TL is linked to chromosome hydroxymethylation patterns.

## 2. Results

### 2.1. Approach Applicability to Telomere Length Analysis in Chorionic Cytotrophoblast Cells

We assessed TL on metaphase chromosomes using Q-FISH, i.e., by measuring fluorescent signal intensity after hybridisation with telomere region-specific DNA probes. The choice of the method was determined by such benefits as its high resolving power and single-cell, individual chromosome, and sister chromatid analysis capabilities. The metaphase plates were obtained from spontaneously dividing chorionic cytotrophoblast cells, which represent the closest possible reflection of in vivo processes. As a result, the applied approach enabled us to rule out deceased cells and those entering apoptosis from the analysis and investigate TL only in viable cells on the same cell cycle stage—the mitosis metaphase.

**Figure 1:**
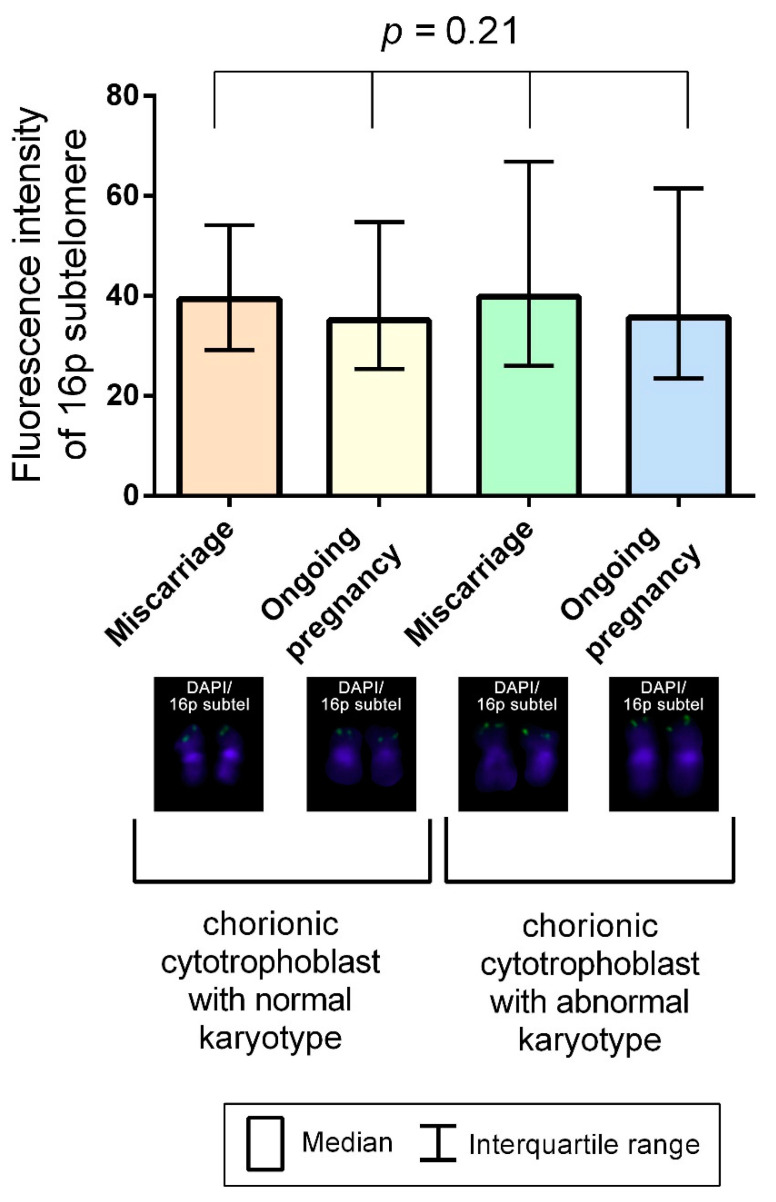
Column bar charts of 16p subtelomeric fluorescence intensity in the metaphase chromosomes from chorionic cytotrophoblast cells in karyotypically normal and abnormal miscarriages and ongoing pregnancies. The Kruskal–Wallis test showed no significant difference among the groups (*p* = 0.21).

To mitigate the impact of chromosome condensation levels, which vary across the metaphase plates on the preparation, on measurement results, we calculated relative TLs. To that end, we divided absolute telomere fluorescence values on each metaphase plate to the fluorescence level of the reference region—in our case, the subtelomeric region of the short arm of chromosome 16 (16p), which is characterised by an exceptionally low, if at all present, interindividual variability. The Kruskal–Wallis test showed no significant difference (*p* = 0.21) in 16p subtelomere’s fluorescence intensity for the metaphases from chorionic cytotrophoblast cells in karyotypically normal and abnormal miscarriages and ongoing pregnancies (Figure 1).

Metaphase plates in cytogenetic preparations from spontaneously dividing cells vary considerably in quality and information value: some experience random chromosome losses, while others feature overlapping chromosomes. To eliminate the impact of these factors, we measured telomere fluorescence signals on the same easily-identified chromosome in every metaphase plate—chromosome 16. The prominent feature of chromosome 16 homologues is a large heterochromatic region in 16q. The reliable identification of chromosome 16 was also enabled by FISH signal in 16p subtelomeric region.

**Figure 2:**
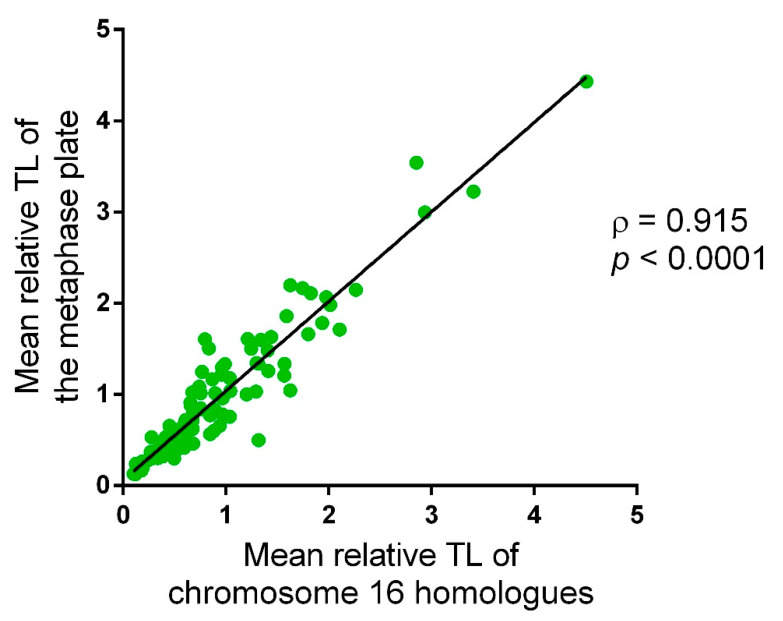
The correlation between the mean relative telomere length (TL) of chromosome 16 homologues and that of other chromosomes in the same metaphase assessed in 107 metaphases across 10 chorionic cytotrophoblast samples (Spearman test, ρ = 0.915; *p* < 0.0001).

We performed correlation analysis to ensure that the relative TL in chromosome 16 reflects the mean relative TL in all chromosomes on a metaphase plate. The analysis employed the following algorithm: In 107 metaphase plates, we measured the intensity of telomeric DNA probe fluorescent signal on the short and on the long arm of each chromatid in every chromosome (four measurements on each chromosome). Then, we added up the obtained values and divided the sum by the number of chromosomes, thus determining the mean value for each metaphase plate. We also calculated the mean fluorescence intensity for the reference DNA probe on each metaphase plate. Next, we calculated the fluorescence intensity of the DNA probes to 16p subtelomeric region on each chromatid (two measurements for each chromosome). In the cells disomic for chromosome 16, we performed calculations on two chromosome 16 homologues, and in those trisomic for chromosome 16, on three homologues. For each metaphase plate, we calculated a ratio between the mean telomeric DNA probe fluorescence intensity and the mean 16p subtelomeric DNA probe fluorescence intensity. We performed a regression analysis of the correlation between the values calculated for chromosome 16 homologues and those for the entire chromosome set of a metaphase plate. The Spearman rank correlation test showed a strong positive correlation between these parameters (ρ = 0.915, *p* < 0.0001) (Figure 2), attesting to the possibility of using the mean relative fluorescence intensity of telomeric DNA probes in chromosome 16 homologues for TL assessment of the whole chromosome set in a cell.

### 2.2. A Comparison of Telomere Lengths in Chorionic Cytotrophoblast from Karyotypically Normal and Abnormal Miscarriages and Ongoing Pregnancies

**Figure 3:**
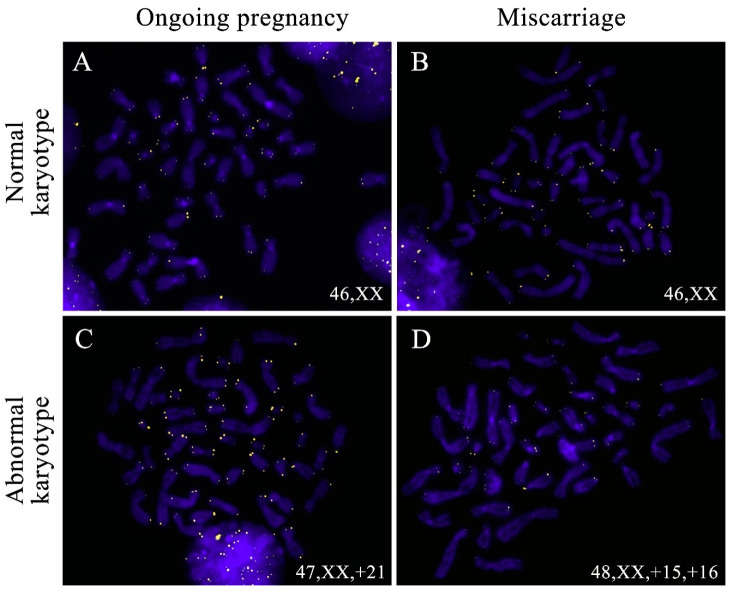
Telomere detection in the metaphase chromosomes from chorionic cytotrophoblast cells in karyotypically normal (**A**,**B**) and abnormal (**C**,**D**) miscarriages (**B**,**D**) and ongoing pregnancies (**A**,**C**). Telomeres were detected through fluorescent in situ hybridisation (FISH) with telomeric DNA probes, and the chromosomes were stained with DAPI.

Our study enrolled chorion samples from first-trimester miscarriages and ongoing pregnancies. Both groups contained karyotypically normal and abnormal samples. For inter-group comparison, we evaluated mean relative TLs of chromosome 16 homologues in 12 metaphase plates in each sample totaling to 6236 values across the groups (Figure 3).

The karyotypically abnormal chorion samples from miscarriages comprised varying numerical chromosomal abnormalities, including trisomy 21, trisomy 16, trisomy 9, monosomy X, double trisomy 15 and 16 and triploidy. Meanwhile, the karyotypically abnormal chorion samples from ongoing pregnancies were all trisomic for chromosome 21. Therefore, to verify whether the comparison between the groups is reasonable, we first compared mean relative TLs measured in the trisomy 21 samples to those measured in the samples with other karyotype abnormalities within the group of karyotypically abnormal miscarriages. The Mann–Whitney U test showed no significance (*p* = 0.15).

**Figure 4:**
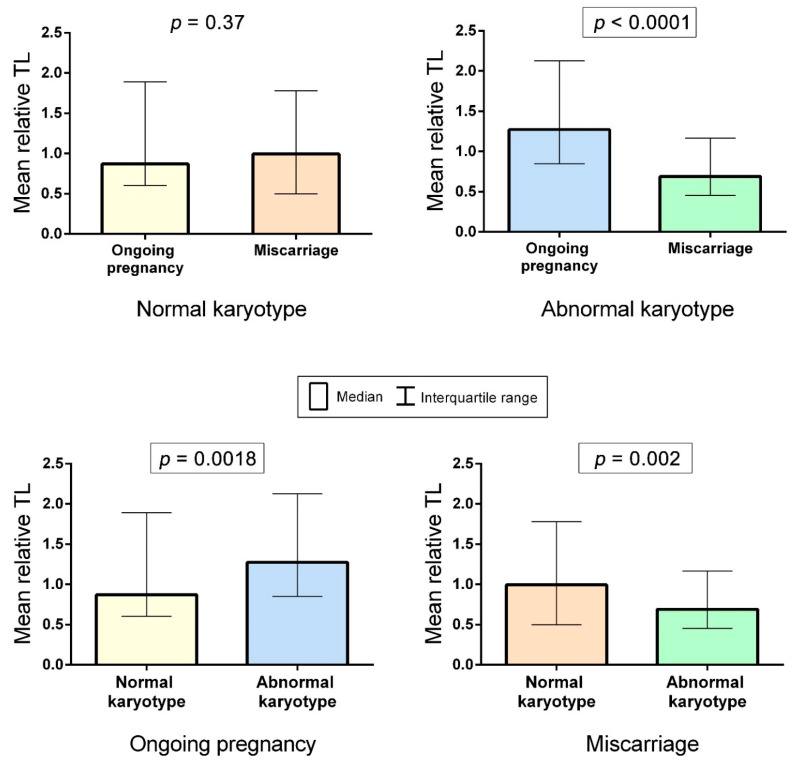
Mean relative telomere lengths (TLs) in metaphase chromosomes from chorionic cytotrophoblast in karyotypically normal and abnormal miscarriages and ongoing pregnancies. The comparisons showing significant difference (the Mann–Whitney U test) are framed.

We compared mean relative TLs between the karyotypically normal samples from miscarriages and ongoing pregnancies. The Mann–Whitney U test showed no significance (*p* = 0.3739) (Figure 4). However, among the karyotypically abnormal chorion samples, mean relative TLs appeared to be significantly higher in ongoing pregnancies compared to miscarriages (*p* < 0.0001) (Figure 4). Moreover, when comparing mean relative TLs between chorion samples from karyotypically normal and abnormal ongoing pregnancies, we revealed significantly higher values in the abnormal karyotype cases (*p* = 0.0018) (Figure 4). In miscarriages, we observed the opposite picture: Mean relative TL values were higher in the karyotypically normal chorion samples (*p* = 0.002) (Figure 4). Therefore, our findings suggest a link between the mean relative TL in metaphase chromosomes from chorionic cytotrophoblast and both developmental capacity and normal/abnormal karyotype of the embryo.

**Figure 5:**
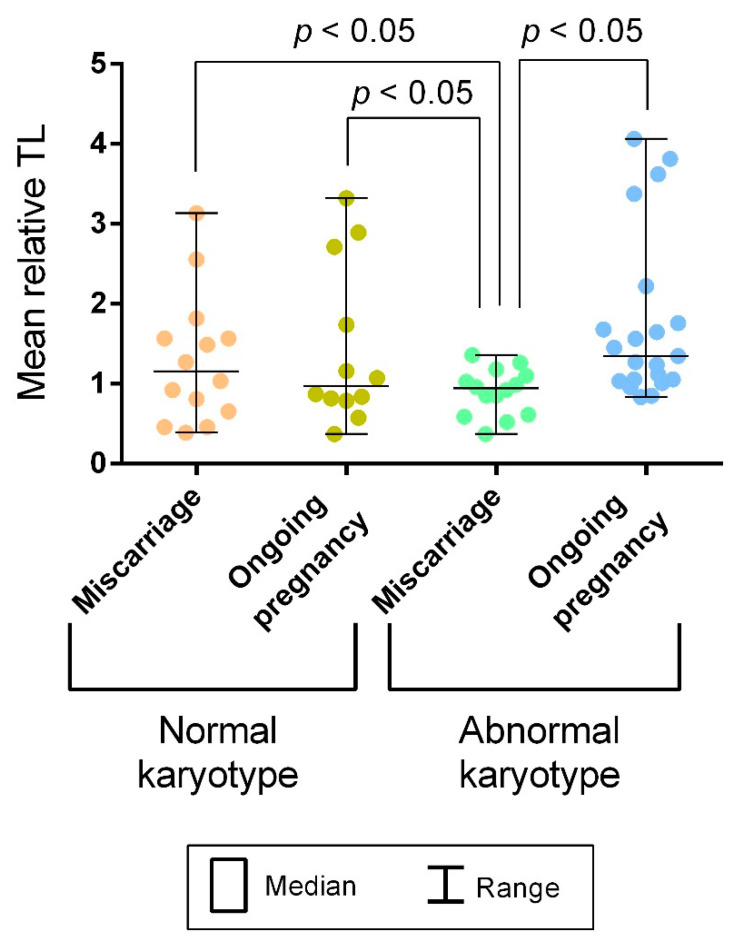
Interindividual variability of mean relative telomere lengths (TLs) in metaphase chromosomes from chorionic cytotrophoblast in karyotypically normal and abnormal miscarriages and ongoing pregnancies. In karyotypically abnormal chorionic cytotrophoblast the TL variance is significantly lower than in other groups under study (*p* < 0.05).

Further on, considering that TL may vary across individuals, we assessed its interindividual variability. Using Levene’s test, we compared variances of the mean relative TLs calculated for each sample among the groups. The variances did not differ across the three groups of samples: karyotypically normal chorionic cytotrophoblast from miscarriages, karyotypically normal chorionic cytotrophoblast from ongoing pregnancies and karyotypically abnormal chorionic cytotrophoblast from ongoing pregnancies (*p* = 0.92 for karyotypically normal miscarriages and ongoing pregnancies; *p* = 0.14 for karyotypically normal and abnormal ongoing pregnancies). However, in all three groups, the variance was significantly higher than in karyotypically abnormal chorionic cytotrophoblast from miscarriages (*p* < 0.05) (Figure 5). Consequently, in all of the groups except the chorionic cytotrophoblast from karyotypically abnormal miscarriages, mean relative TLs varied widely.

### 2.3. Interchromatid Telomere Length Asymmetry in Chorionic Cytotrophoblast and Adult Lymphocytes

A fascinating phenomenon drew our attention as we assessed TLs in chorionic metaphase chromosomes: the size of telomeric FISH signals differed between sister chromatids.

To investigate interchromatid telomere differences among the examined groups, we calculated TL ratios between sister chromatids in chromosome 16 by dividing the higher telomere fluorescence intensity by the lower. Two values were calculated for each chromosome 16: the ratio of TLs in the sister chromatids in the short chromosome arms and that in the long chromosome arms. A total of 669 and 870 values were obtained for chorion samples of karyotypically normal and abnormal miscarriages, respectively, and 553 and 998 values for chorion samples of karyotypically normal and abnormal ongoing pregnancies, respectively. The D’Agostino and Pearson omnibus normality test showed that values in all of the groups were not normally distributed. Using the Kruskal–Wallis test, we compared the obtained values, which reflected an interchromatid TL asymmetry, in all of the examined groups without any significant difference between them (*p* = 0.22).

**Figure 6:**
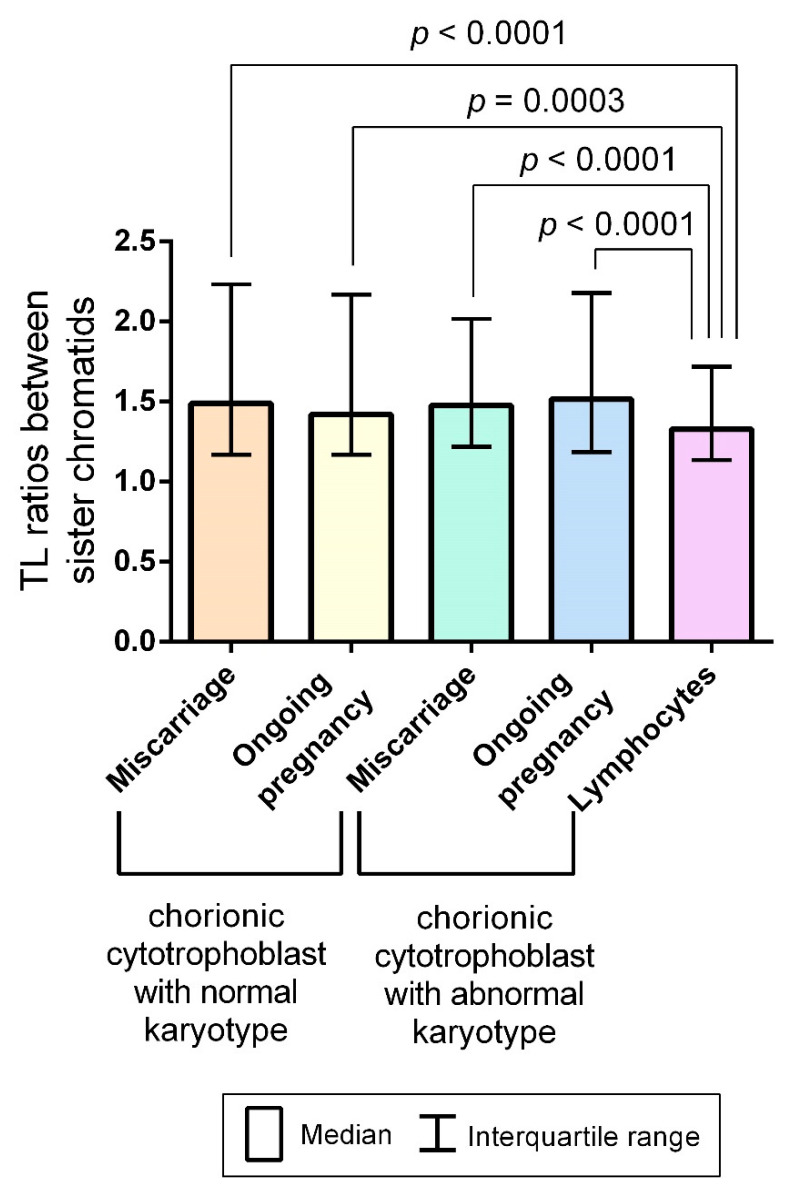
Column bar charts of telomere length (TL) ratios between the sister chromatids of chromosomes 16 in the chorionic cytotrophoblast from miscarriages and ongoing pregnancies and in PHA-stimulated peripheral blood lymphocytes of healthy adults. TL ratios do not differ among karyotypically normal and abnormal chorionic cytotrophoblast samples from miscarriages and ongoing pregnancies (the Kruskal–Wallis test, *p* = 0.22). Still, they are significantly higher than those in PHA-stimulated lymphocytes (the Mann–Whitney U test, *p* < 0.0001, *p* = 0.0003).

TL asymmetry in the mother and daughter chromatids could manifest a recombination-based mechanism of telomere lengthening ALT during active mitotic divisions of chorionic cytotrophoblast cells. Therefore, we deemed it rational to compare interchromatid TL ratios measured in spontaneous mitoses in each group of the chorion samples to those measured in PHA-stimulated lymphocytes (a total of 449 values). The Mann–Whitney U test showed higher interchromatid TL ratios in all four of the examined groups of chorion samples compared with those in PHA-stimulated lymphocytes (*p* < 0.0001 for comparison with karyotypically normal and abnormal miscarriages and karyotypically abnormal ongoing pregnancies and *p* = 0.0003 for comparison with karyotypically normal ongoing pregnancies), thereby suggesting a more pronounced interchromatid TL asymmetry in the chorionic cytotrophoblast (Figure 6).

### 2.4. Interchromatid Telomere Length Asymmetry in Chorionic Cytotrophoblast Is Linked to DNA Hydroxymethylation Pattern

TL regulation is considerably influenced by epigenetic mechanisms [35,36,37,38]. In contrast to the well-researched regulation of telomeres by DNA methylation, the link between TL and 5-hydroxymethylcytosine (5hmC), an oxidative derivative of 5-methylcytosine, remains much less elucidated. Recent studies have demonstrated inter-chromosome and inter-cell variability of 5hmC patterns in cultured [39,40,41] and noncultured human cells, including the chorionic cytotrophoblast [42]. One intriguing feature of this variability is the asymmetrical pattern of hydroxymethylation in some metaphase chromosomes with 5hmC in only one sister chromatid—hemihydroxymethylation [39,40,41,42].

**Figure 7:**
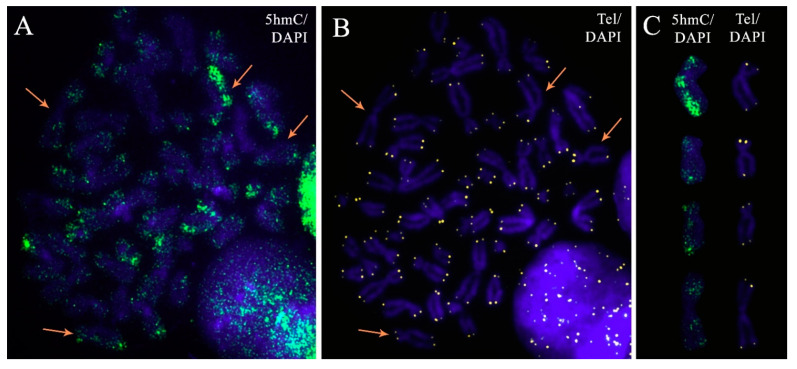
The metaphase plate from a chorionic cytotrophoblast cell after immunocytochemical detection of 5-hydroxymethylcytosine (5hmC) (**A**) and detection of telomeric regions through fluorescent in situ hybridisation (FISH) with telomeric DNA probes (**B**). The chromosomes were stained with DAPI. The arrows show chromosomes represented on karyogram (**C**): those demonstrating the asymmetrical pattern of hydroxymethylation with 5hmC in only one sister chromatid—hemihydroxymethylation.

Considering chromosome hemihydroxymethylation and the discovery of interchromatid TL asymmetry in the present study, we investigated the possible link between these phenomena. We performed immunocytochemical detection of 5hmC on the metaphase preparations analysed for TLs. Then, we identified hemihydroxymethylated chromosomes and calculated the TL ratios between sister chromatids by dividing the telomere fluorescence in the 5hmC-positive (5hmC+) chromatid by the telomere fluorescence in the 5hmC-negative (5hmC-) chromatid (Figure 7). A total of 2288 values were obtained across the examined groups: 238 and 1110 in the karyotypically normal and abnormal ongoing pregnancies, respectively, and 494 and 446 in the karyotypically normal and abnormal miscarriages. The D’Agostino and Pearson omnibus normality test showed an abnormal distribution of values in all of the groups (*p* < 0.0001). The Kruskal–Wallis test showed no statistically significant difference among the groups (*p* = 0.68), and in further calculations, all of the 2288 obtained values were treated as a single sample.

The median value for TL ratios between 5hmC+ and 5hmC-sister chromatids was 1.029. The one-sample Wilcoxon signed-rank test showed that it significantly exceeded “1.0” (*p* < 0.0001), which is the expected value for the ratio of equal TLs in 5hmC+ and 5hmC- sister chromatids. Therefore, in hemihydroxymethylated chromosomes, 5hmC+ sister chromatid predominantly contains longer telomere than 5hmC- one.

## 3. Discussion

In the first trimester of gestation, human embryo development is marked by an intensive growth of extraembryonic tissues, including chorion, which plays a crucial role in facilitating maternal–foetal interactions. The proliferative capacity of chorionic cells, such as that of any other mammalian cells, is determined by TL. It is evident that mitotically active cells require telomere lengthening to ensure normal growth and functioning of extraembryonic tissues throughout the pregnancy. The ample available evidence suggests the presence of telomerase in extraembryonic tissues and a physiological decrease in its activity in the placenta as the pregnancy progresses [43,44,45]. However, normal telomerase expression and activity in placental tissues is too low to maintain telomere homeostasis for the entire gestation period. The alternative mechanism of telomere elongation–ALT–which is based on recombination, has not been directly observed in gestational tissues [46]. Nevertheless, in first-trimester trophoblasts, a higher TERRA expression than in matched somatic cells from cord blood coincides with extremely low telomerase levels, suggesting a possible ALT role [47]. Interchromatid TL asymmetry detected in metaphase chromosomes from chorionic cytotrophoblast cells in the present study also indirectly suggests that telomere lengthening may involve recombination between the homologous regions of telomeric DNA. A lower level of interchromatid telomere asymmetry in lymphocytes as compared with that in chorionic cytotrophoblast cells (Figure 6) attests to a potential association of this phenomenon with cell proliferative activity.

The obtained results also demonstrate a relationship between interchromatid TL asymmetry and chromosome 5hmC patterns: the longer telomere was predominantly present in the hydroxymethylated sister chromatid, while the non-hydroxymethylated sister chromatid mostly featured a shorter telomere. Recent studies on mouse embryonic stem cells, human blood cells and head and neck squamous cell carcinomas have demonstrated the role of TET hydroxylases and 5hmC in the maintenance of telomere homeostasis, including that occurring through telomere recombination [48,49,50,51]. In our recent study on chromosomes from human cytotrophoblast cells, an immense heterogeneity of 5hmC patterns has been demonstrated: different types of chromosomal 5hmC accumulation—hydroxymethylation, hemihydroxymethylation and nonhydroxymethylation with no specificity of each accumulation type to any particular chromosome in a given metaphase plate [42]. In combination with the findings of the present study, the abovementioned results may suggest that, firstly, 5hmC marks the sister chromatid with a longer telomere that may further serve as a template for telomere elongation through ALT, and secondly, that it potentiates a recombination process through establishing specific chromatin states. Judging by the “chaotic” distribution of hemihydroxymethylated chromosomes, any chromosome in a given metaphase plate could be marked with 5hmC for telomere recombination. Coupled with high mitotic activity, this would ensure the maintenance of TLs across the tissue, even if the process did not involve every chromosome after each replication cycle. Considering the susceptibility of 5hmC patterns to environmental stimuli [52], the detected relationship between interchromatid TL asymmetry and chromosome hydroxymethylation patterns may also suggest a possible pathway to telomere regulation by external factors including adaptive response to stress [53].

Interchromatid telomere asymmetry discovered in the present study and its link to 5hmC patterns were typical for cycling chorionic cytotrophoblast cells both in ongoing and arrested pregnancies and did not depend on the presence of karyotype abnormalities. Therefore, these phenomena, which are potentially associated with ALT, could be integral to the development programme. However, when comparing TLs in chorionic cytotrophoblasts from karyotypically normal and abnormal miscarriages and ongoing pregnancies, we detected differences between the groups (Figure 4) and a pronounced interindividual TL variability (Figure 5). These findings may be explained by a leading role for heredity, not the mechanisms of telomere elongation, in determining TL in chorionic cytotrophoblast. The heritability of TLs has been already demonstrated on the lymphocytes of monozygotic and dizygotic twins [54,55,56], fibroblasts and amniocytes [55] and zygotes [57]. Consequently, TL is likely to be already determined when an organism starts its development. Further on, embryos undergo robust negative selection resulting in the arrest of those with low developmental capacity. Having found longer telomeres in karyotypically abnormal chorionic cytotrophoblast from ongoing pregnancies compared with those from miscarriages, we may assume that TL strongly impacts the viability of karyotypically abnormal embryos. Furthermore, telomeres were longer in karyotypically abnormal chorion from ongoing pregnancies than in karyotypically normal matched samples (Figure 4), and the lower limit of TL variability in the former group was higher than in other investigated groups (Figure 5). The inheritance of longer telomeres may promote the development of a karyotypically abnormal embryo, while a combination of shorter telomeres with a chromosomal pathology, even one compatible with life, leads to early miscarriage. These suggestions provide further support to a study by Huleyuk and colleagues, demonstrating short telomeres in spontaneously eliminated aneuploid embryos [58], and the discovery of longer telomeres in newborns with trisomy 21 compared with karyotypically normal ones [59,60]. In karyotypically normal pregnancies, by contrast, TL does not seem to considerably affect developmental capacity and is obviously not among major contributors to sporadic euploid pregnancy loss as we observed, similar to TL itself and its variation range in the karyotypically normal chorionic cytotrophoblast from miscarriages and that from ongoing pregnancies (Figure 4 and Figure 5).

The limitations of the present study related to the using of metaphase Q-FISH for TL analysis should be noted. The analysis was performed exclusively on the direct preparations of metaphase plates from chorionic villi, i.e., on spontaneously dividing cells. On the one hand, this approach enabled us to investigate TL only in proliferating chorionic cytotrophoblast cells, which closely reflects the situation *in vivo*, and made possible the analysis of TLs in individual chromosomes and sister chromatids. On the other hand, the number of obtained metaphases on the direct preparation is relatively low, which restricts the sample size. Manual evaluation of fluorescence signals on metaphase chromosomes allows accurate analysis and exclusion of background noise, but also restricts the possible number of measurements. Finally, due to Q-FISH resolution limits, signals at telomeric repeats below the threshold for the PNA probe hybridisation can be missed.

To conclude, our results suggest that interchromatid TL asymmetry and its association to 5hmC patterns in chorionic cytotrophoblast—two phenomena potentially linked to the ALT—are integral to the development programme. The central role in determining TL belongs to heredity. The inheritance of long telomeres by a karyotypically abnormal embryo promotes his development. By contrast, in karyotypically normal first-trimester embryos, TL does not seem to have a considerable impact on developmental capacity. Our results contribute to the knowledge of telomere biology in human embryogenesis and open new directions for investigating 5hmC regulation of telomere lengthening.

## 4. Materials and Methods

### 4.1. Sample Collection

The chorionic villi were obtained at the D.O. Ott Research Institute of Obstetrics, Gynecology and Reproductology from 61 patients: those who were diagnosed with first-trimester missed abortion and underwent dilation and curettage (*n* = 28, the miscarriage group) and those with ongoing first-trimester pregnancy who underwent chorionic villus sampling (CVS) for prenatal genetic diagnosis (*n* = 33, the ongoing pregnancy group). The chorionic villi were dissected under the Leica M125 stereomicroscope and rinsed from blood clots in 0.9% NaCl.

The miscarriage group included 14 karyotypically normal and 14 karyotypically abnormal samples. The latter included trisomy 16 (*n* = 6), trisomy 21 (*n* = 3), trisomy 9 (*n* = 1), monosomy X (*n* = 1), double trisomy 15 and 16 (*n* = 1) and triploidy (*n* = 2). The ongoing pregnancy group included 12 karyotypically normal (the indications for the CVS were monogenic diseases) and 21 karyotypically abnormal samples (the indications for the CVS were ultrasonic and biochemical chromosomal pathology markers). All of the karyotypically abnormal samples were trisomic for chromosome 21.

The peripheral blood lymphocytes were obtained from 11 adult, karyotypically normal volunteers.

### 4.2. Chromosome Preparation

Metaphase chromosomes from the chorionic cytotrophoblast were prepared by the direct technique (without culturing) according to a protocol with modifications described previously [61].

Metaphase chromosomes from the lymphocytes were prepared after the culturing of blood samples for 72 h at +37 °C, with phytohemagglutinin (PHA) (M021, PanEco, Moscow, Russia) added into the culture medium. Metaphase harvesting, hypotonic treatment, fixation and chromosome spreading on the slides were performed according to the standard techniques with minor modifications previously used in our laboratory [62].

### 4.3. Fluorescence In Situ Hybridisation (FISH)

Telomeric regions were detected on the chromosome preparation slides through fluorescence in situ hybridisation (FISH) with telomeric probes (K532611-8, DAKO, Glostrup, Denmark). All of the procedures were performed in line with the manufacturer’s recommendations, with minor modifications described previously [57].

After the photoimaging, 16p subtelomeric region (the reference region for TL measurements in our study) was detected on the same metaphase preparations through FISH with a TelVysion SpectrumGreen 16p DNA probe (05J03-016, Abbott Laboratories, Chicago, IL, USA).

### 4.4. Immunodetection of 5-Hydroxymethylcytosine (5hmC)

5hmC was detected on the metaphase chromosome preparations through immunofluorescence with primary antibodies against 5hmC (39769, Active Motif, Carlsbad, CA, USA) and secondary goat anti-rabbit Alexa Fluor 488 (A11008, Life Technologies, Carlsbad, CA, USA) antibodies according to the protocol used in earlier studies [63,64]. The DNA denaturation step was excluded from the immunofluorescence protocol because it was carried out earlier during FISH procedures.

### 4.5. Image Acquisition and Evaluation of Telomeric and Subtelomeric FISH-Signal Intensity

The fluorescence images of metaphases after FISH and 5hmC immunodetection were recorded using the Leica DM 2500 microscope, the Leica DFC345 FX camera and the Leica Application SuiteV.3.8.0 software. The following acquisition options were used for the images of chromosomes after hybridisation with telomeric DNA probes: exposure time—1.8 s, gain—x6, gamma—3.44; after hybridisation with the 16p DNA probe: exposure time—2.0 s, gain—x2, gamma—2.0.

The telomeric and subtelomeric FISH-signal size and intensity were evaluated on the digital photoimages using the Image J 1.49v software. The fluorescence intensity was measured in relation to the signal area using the Freehand Selection tool through manual selection of each signal area.

### 4.6. Statistical Analysis

Statistical analysis was carried out with GraphPad Prism (Version 6.01, GraphPad Software Inc., San Diego, CA, USA). Normality was checked using the D’Agostino–Pearson omnibus normality test. The inter-group comparisons were carried out using the Mann–Whitney U test and the Kruskal–Wallis test. The variances were compared in IBM SPSS Statistics (Version 23, IBM, Armonk, NY, USA) using Levene’s test. The α-level was set at 0.05.
